# Targeting the T-Lak cell originated protein kinase by OTS964 shrinks the size of power-law coded heterogeneous glioma stem cell populations

**DOI:** 10.18632/oncotarget.23077

**Published:** 2017-12-09

**Authors:** Michiya Sugimori, Yumiko Hayakawa, Masaki Koh, Tomohide Hayashi, Ryoi Tamura, Satoshi Kuroda

**Affiliations:** ^1^ Department of Integrative Neuroscience, University of Toyama, 2630 Sugitani, Toyama, Toyama 930-0194, Japan; ^2^ Department of Neurosurgery, University of Toyama, 2630 Sugitani, Toyama, Toyama 930-0194, Japan

**Keywords:** glioma stem cell (GSC), glioma sphere (GS), T-Lak cell originated protein kinase (TOPK), OTS964, power-law

## Abstract

Glioblastoma resists chemoradiotherapy, then, recurs to be a fatal space-occupying lesion. The recurrence is caused by re-growing cell populations such as glioma stem cells (GSCs), suggesting that GSC populations should be targeted. This study addressed whether a novel anti-cancer drug, OTS964, an inhibitor for T-LAK cell originated protein kinase (TOPK), is effective in reducing the size of the heterogeneous GSC populations, a power-law coded heterogeneous GSC populations consisting of glioma sphere (GS) clones, by detailing quantitative growth properties. We found that OTS964 killed GS clones while suppressing the growth of surviving GS clones, thus identifying clone-eliminating and growth-disturbing efficacies of OTS964. The efficacies led to a significant size reduction in GS populations in a dose-dependent manner. The surviving GS clones reconstructed GS populations in the following generations; the recovery of GS populations fits a recurrence after the chemotherapy. The recovering GS clones resisted the clone-eliminating effect of OTS964 in sequential exposure during the growth recovery. However, surprisingly, the resistant properties of the recovered-GS clones had been plastically canceled during self-renewal, and then the GS clones had become re-sensitive to OTS964. Thus, OTS964 targets GSCs to eliminate them or suppress their growth, resulting in shrinkage of the power-law coded GSC populations. We propose a therapy focusing on long-term control in recurrence of glioblastoma via reducing the size of the GSC populations by OTS964.

## INTRODUCTION

Cancer contains a cell population that initiates a tumor [1–3]. An initiating cell population produces cancer cells, while maintaining/expanding the population size [2, 4]. This can result from “cancer stem cells (CSCs)”, and thus cancer can be considered an abnormal developmental organ with exaggerated plastic functions of CSCs [1]. The CSC population is also thought to be responsible for the regrowth of the tumor after chemoradiotherapies, suggesting that the population should be targeted for controlling tumor recurrence [5–8]. This proposes a strategic challenge in order to reduce the size of CSC populations via regulating CSC’s plastic functions [2, 9]. However, how CSCs qualitatively and quantitatively maintain/grow the size of their populations remains to be determined [2, 7, 9–11].

Glioblastoma is a malignant brain tumor that resists combined chemoradiotherapies, and then recurs. The recurrence provides little hope for patients [6, 11]. Identification of CSCs in glioblastoma, namely glioma stem cells (GSCs), provides a potential treatment strategy where GSCs can be specifically targeted [12, 13]. However, heterogeneity in CSC/GSCs raises another challenge, namely to understand how well qualitatively and quantitatively coordinated heterogeneous CSC/GSC populations self-renew [4, 9, 14, 15]. We recently found that GSC populations are functionally heterogeneous in growth by following a power-law; specifically, that the power-law coded heterogeneous GSC populations self-renew [9]. The power-law phenomenon explains how well the heterogeneity is plastically organized, and how robust the heterogeneity is maintained in the self-renewal of GSC populations. However, it still remains unexplored whether GSCs exhibit another functional heterogeneity; how GSCs quantitatively self-renew while exhibiting functional heterogeneity in the power-law growth. These questions should be answered by identification of the functional characteristics at the CSC/GSC population level.

One of the major issues in chemoradiotherapies is sensitivity/resistance of tumors. Since there would be heterogeneity in sensitivity/resistance of tumors, resistant cells/clones would contribute to re-grow and consequently cause a tumor recurrence [16], suggesting that sensitivity/resistance should be considered as important functions of GSCs. While the qualitative nature of this process is appreciated, it is important to reveal how quantitatively GSC populations exhibit heterogeneity in sensitivity/resistance to chemoradiotherapies [2, 8, 17].

When we previously administered the anti-glioblastoma/GSC agent Temozolomide (TMZ) to U87-derived power-law coded GS populations, we found shrinkage of the size of the population while maintaining significant survival of self-renewable GSC clones coded by a power-law. A removal of TMZ allowed a power-law coded growth recovery of the surviving GSC clones suggesting that TMZ never collapsed the heterogeneous GSC populations. We thus have proposed a hypothesis where a collapse of scale-free power-law may cause disruptions of heterogeneous GSC populations [9], however, how to collapse a power-law has not yet been determined.

In this study, we investigated the efficacy of a novel anti-cancer drug for T-lymphokine-activated killer cell originated protein kinase (TOPK; also known as PBK or PDZ-binding kinase; HGNC: 18282) OTS964, which selectively inhibits kinase activity of TOPK with high affinity in the self-renewal of power-law coded heterogeneous GSC populations [18]. TOPK, a Ser/Thr protein kinase, is expressed and trans-activated in broad types of human cancer such as lung and breast cancers [19–22]. TOPK is also listed as a “consensus stemness ranking signature” gene as expressed in CSC enriched tumors [23], thus, it was hypothesized that an inhibitor for targeting TOPK would be promising treatment for tumors with frequent recurrence from CSCs. TOPK is suggested to be involved in the growth of glioblastoma and of GSCs [24, 25] (Hayashi *et al.*, in press). Another inhibitor of TOPK, HITOPK-032, down-regulated the growth and survival of GSCs [25]; OTS964 is effective for many cancers in both *in vitro* and *in vivo* xenograft models [18, 26]. However, it still remains unclear whether OTS964 is effective in power-law coded heterogeneous GSC populations that show sensitivity/resistance. We thus sought to identify whether and how OTS964 affects the size of power-law coded GSC populations, and to show functional heterogeneity in sensitivity/resistance to OTS964 in the self-renewal of heterogeneous GSC populations by taking advantage of a quantitative approach, clonal tumor neurosphere culture.

## RESULTS

### Dose dependent reduction in the size of GS populations by OTS964

U87 and U251 glioma cells significantly expressed TOPK (Supplementary Figure 1A and 1B). The viability of U87 and U251 decreased in proportion to various concentrations of OTS964 (Supplementary Figure 1C and 1D).

**Figure 1:**
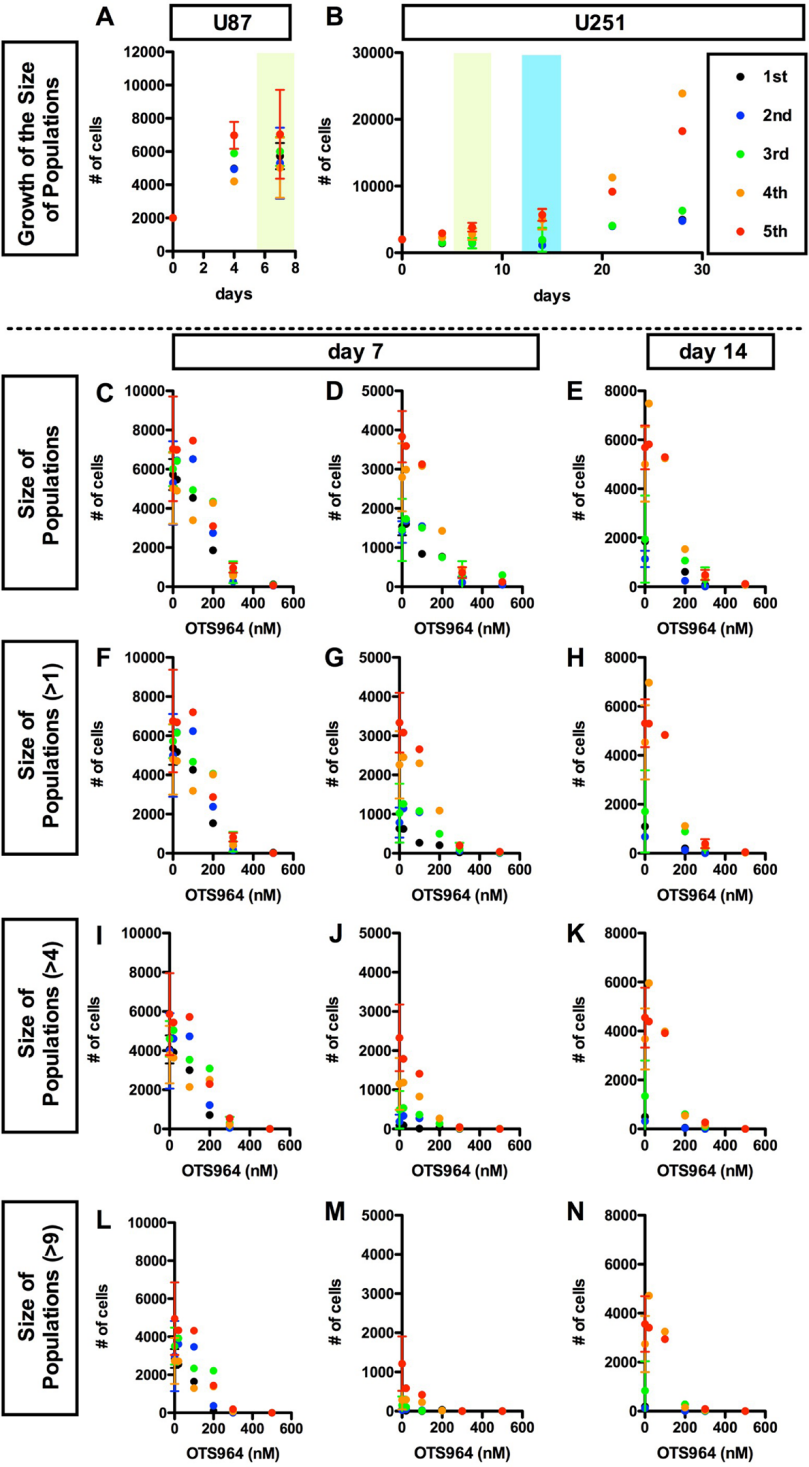
OTS964 disturbed the growth of the size of glioma stem cell populations in a dose dependent manner We seeded glioma cell line-derived cells in methylcellulose-containing growth medium at an initial clonal density of 4,000 cells/2 ml in each well of 12 well-plates. Each clone differentially grew and some became glioma spheres: GSs. A and B, Consistent growth in the size of U87 (**A**) or U251 (**B**) -derived GSC population in the methylcellulose-containing growth media. The size of an entire glioma sphere (GS)-forming cell population was calculated as the summation of number of cells of all clones, or as the product of multiplication of the average number of cells/clone by the number of survived clones. The size, as number of cells in an entire population, is shown at each time point (0, 4, 7 days for A and B; 14, 21, 28 days for B, respectively). The data for 5 generations are shown (1st shown in black filled circles; 2nd: blue; 3rd: green; 4th: orange; 5th: red, respectively). The cell populations were consistently grew during cultured period at every generation. C-N, Dose dependent suppression in the growth of the GS population in the presence of OTS964. The sizes of the cell population of all clones (**C**–**E**); of self-renewed clones (**F–H** for clones with multiple cells); of expanded clones (**I**–**K** for clones with more than 4 cells; **L–N** for clones with more than 9 cells) are shown at various concentrations of OTS964 (0, 20, 100, 200, 300 and 500 nM). The graphs are for U87- (C, F, I and L) and for U251-derived (D, E, G, H, J, K, M and N) GS clones. The data of 20, 100 and 500 nM were taken from the first series of experiments; 200 nM, the second; 300 nM, the third; and the control (0 nM) from the first through the fourth series. OTS964 disturbed the growth of the size of GS-forming cell population, and the effects were dose-dependent.

**Figure 2:**
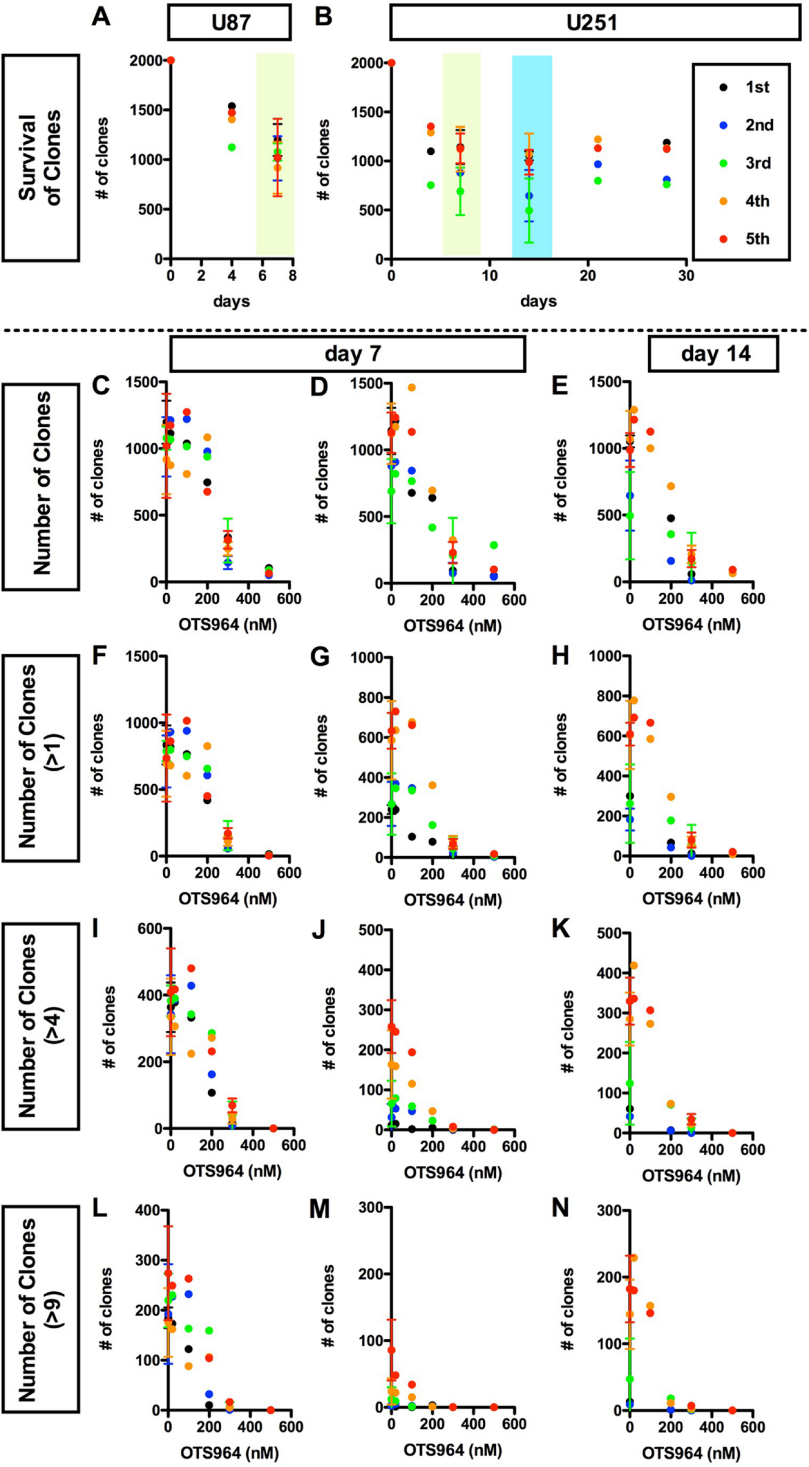
Dose dependent disturbance in the survival of clones by OTS964 in glioma stem cell populations (**A** and **B**), Consistent survival of U87 (A) or U251 (B) -derived GSC clones in the methylcellulose-containing growth media throughout the culture period. The numbers of survived clones are shown at each time point (0, 4, 7 days for A and B; 14, 21, 28 days for B, respectively). The data for 5 generations are shown (1st shown in black filled circles; 2nd: blue; 3rd: green; 4th: orange; 5th: red, respectively). Clones remained to survive consistently during the assayed periods at every generation. C-N, Dose dependent suppression in the survival of clones in the presence of OTS964. The numbers of clones in the population of all clones (**C**–**E**); of self-renewed clones (**F–H** for clones with multiple cells); of expanded clones (**I**–**K** for clones with more than 4 cells; **L**–**N** for clones with more than 9 cells) are shown at various concentrations of OTS964 (0, 20, 100, 200, 300, 500 nM). The graphs are for U87 (C, F, I and L) and for U251 (D, E, G, H, J, K, M and N) -derived GS clones. The data of 20, 100 and 500 nM were taken from the first experiment; of 200 nM, the second; of 300 nM, the third and the fourth and the control (0 nM) from both of the first thorough the fourth. OTS964 suppressed the survival of GS clones, and the effects were dose-dependent.

**Figure 3:**
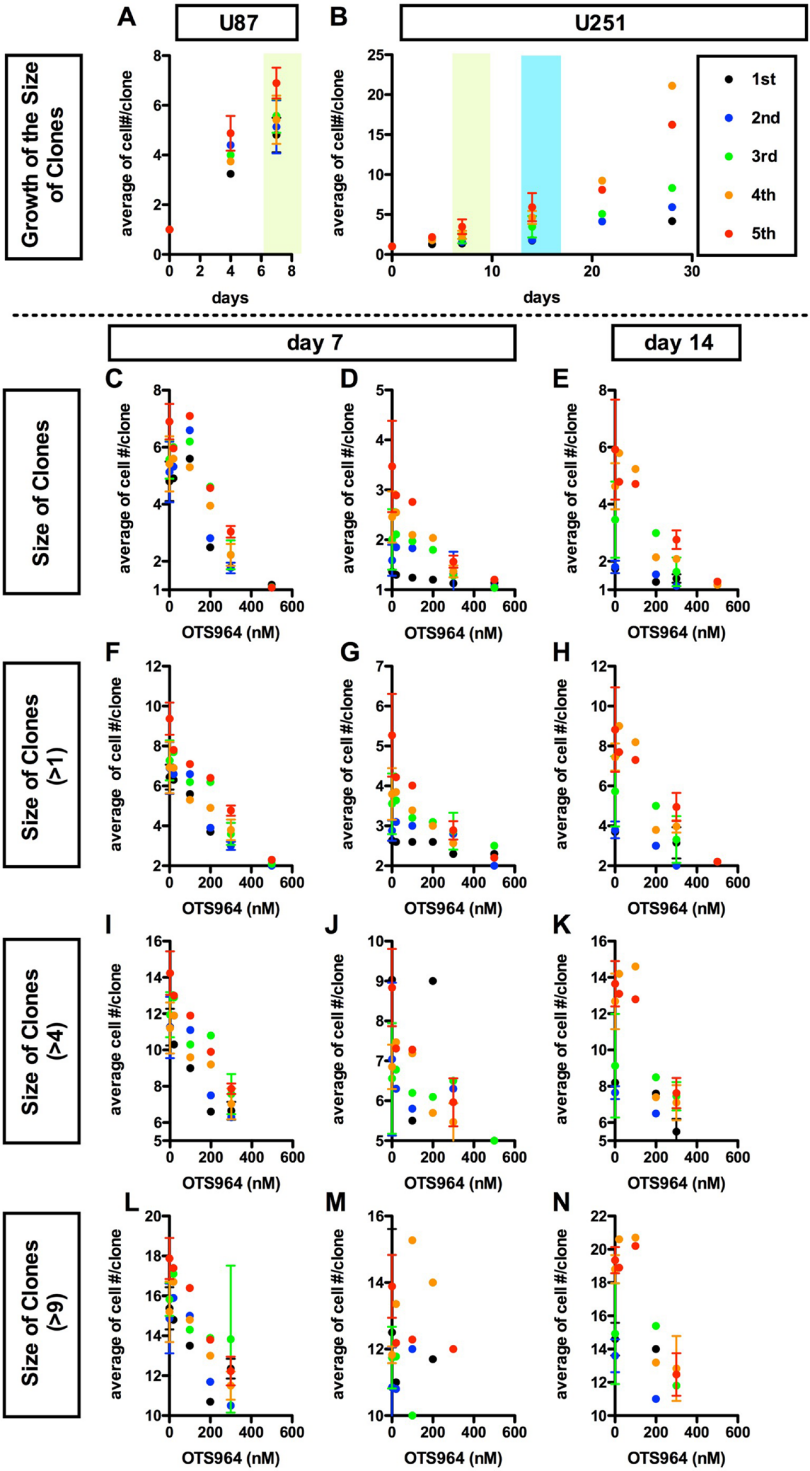
Dose dependent suppression in the average growth of glioma stem cell populations by a TOPK inhibitor OTS964 (**A** and **B**), Consistent growth of U87 (A) or U251 (B) -derived GSC clones in the methylcellulose-containing growth media. The average number of cells/clone is shown at each time point (at 0, 4, 7 days for A and B; 14, 21, 28 days for B, respectively). The experiments were repeated for 5 generations (1st shown in black filled circles; 2nd: blue; 3rd: green; 4th: orange; 5th: red, respectively). (**C**–**N**), Dose dependent suppression in the growth of clones in the presence of a TOPK inhibitor OTS964 (Oncotherapy Science). The average numbers of cells/clone among all clones (C–E); among self-renewed clones (F–H for clones with multiple cells); among expanded clones (I–K) for clones with more than 4 cells; (L–N) for clones with more than 9 cells) are shown at various concentrations of OTS964 (0; 20; 100; 200; 500 nM). The graphs are for U87 (C, F, I and L) and for U251 (D, E, G, H, J, K, M and N) -derived GS clones. The data at 20, 100 and 500 nM were taken from the first experiment; 200 nM from the second; 300 nM from the third and fourth and the control (0 nM) from the first through the fourth, respectively. Existence of OTS964 suppressed the growth of GS clones, and the effects were dose-dependent.

Then, we first tested whether both U87 and U251 consistently expand the size (cell number in a GS population) of GS cell populations at every generation. While we confirmed that U87-derived GS clones grew and recover the population size within a week (Figure 1A) [9], we found that U251-derived GS clones slowly but consistently grew up to recover the original size of GS populations over more than two weeks in the early generations (the 1st to the 3rd generations) (Figure 1B). U251-GS populations, in the late generations (the 4th and further generations), grew more rapidly, and then recovered their population size faster (Figure 1B). In more detail, the survival rates of U87- and U251-derived GS clones were around 50%. The survival rates appeared to be higher than the data in our previous study [9] (see Figure 2). Moreover, the average growth of U87- and later generations of U251-derived GS clones also appeared to be higher than in our previous study (see Figure 3). Consistently, the percentages of single cell clones in U87- and U251-derived GS clones were around 30–40%, suggesting that the GS clones grew in a clonal density. Thus, while both the survival and the growth of GS clones in this study appeared to be enhanced compared to the previous study, we were able to observe consistent growth of the U87- and U251-derived GS populations with maintaining a clonal condition. We then conducted further experiments mainly assaying at day 7 for U87-, and at day 7 and 14 for the late generations of U251-derived GS populations, respectively.

Reduction of tumor size is the primary efficacy for anti-tumor drugs, thus we first addressed whether OTS964 has an efficacy to reduce the size of GS populations. We tried to identify an effective dose for a significant reduction in the U87- and U251-derived GS populations. The values of IC_50_ for U87 and U251 cell lines were about 125 and 68 nM (Supplementary Figure 1C and 1D), respectively, so that we tested five different concentrations, 20, 100, 200, 300 and 500 nM, in the first series of experiments. We found that OTS964 reduced sizes of both the U87- and U251-derived GS populations, and the efficacy was dose dependent (Figure 1C–1E; Supplementary Figure 2B and 2C). Then, we asked whether the size reduction is due to a reduction in larger clone-derived cell populations, because multi-cellular clone-derived cells predominantly occupy the majority of both the U87- and U251-GS populations [9]. The size of GS populations consisted of multi-cellular clones (>1-cell, >4-cell, >9-cell clones) were reduced (Figure 1F–1N) in a similar pattern to the reduction in whole populations (Figure 1C–1E), and the efficacy was dose dependent. This suggests that OTS964 reduced the size of GS populations via shrinking the size of multi-cellular clone-derived populations. Thus, we identified a “size-reducing efficacy” of OTS964 in the growth of the GS populations.

We next asked whether the shrinkage of the size of GS populations in the presence of OTS964 is due to the reduction in survival of GS clones. In this system, both U87- and U251-derived clones consistently survived during the assay period (Figure 2A and 2B; Supplementary Figure 2A), thus, we are able to address the efficacy of OTS964 in the survival of GS clones [9, 27–29]. The number of surviving GS clones of both whole populations and of multi-cellular clones were accordingly reduced, and the reductions were dose dependent (Figure 2C–2N). The dose dependent reducing patterns in the number of surviving GS clones are quite similar to patterns in the shrinkage of the GS populations (Figure 2C–2N; see Figure 1), suggesting that the shrinkage in the GS populations is due to elimination of GS clones. Thus, we identified a “clone-eliminating efficacy” of OTS964 due to suppression of survival of GS clones in the growth of the GS populations.

The size reduction in the GS populations could also be a consequence by the down-regulation of the growth of GS clones [9]. The GS clones consistently grew as shown by an increase in the average cell number in a clone (Figure 3A and 3B; Supplementary Figure 2A). The average number of cells in a clone in both the whole population and multi-cellular clones was reduced in the presence of OTS964, and the dose-dependent reducing patterns appear to be in inverse proportions (Figure 3C–3N). Thus, the dose-dependent reduction in the size of a GS population by OTS964 is also due to the consequence of disturbing the growth of GS clones. Thus, we identified a “growth-disturbing efficacy” of OTS964 in the GS populations. The data above suggest that OTS964 regulates/reduces the size of GSC populations via suppressing survival and disturbing the growth of GS clones (Supplementary Figure 2D).

The efficacy of OTS964 on the GS populations was very significant in the range of hundreds of nM, especially 200 or more nM, for both U87- and U251-derived GS populations. However, 500 nM was too strong to conduct experiments exploring kinetics of “suffered and survived GS clones” after an exposure of OTS964 (Supplementary Figure 3A and 3B), because the population size of the 500 nM experiments was reduced to less than 1% of the controls (0 nM) due to ∼95% elimination of GS clones, while the surviving clones reside as single-cell clones (Figures 1C–1E and 2C–2H). On the other hand, when we administered 300 nM of OTS964, less than 1/3 of the GS clones survived (Figure 2C–2N) but resided as single-cell clones and multi-cellular clones (Figures 2C–2N and 3C–3N). Then, we were able to collect enough cells to conduct further repopulation experiments. Thus, we defined 300 nM as the concentration for further experiments to explore the heterogeneity in sensitivity/resistance and in the recovery/self-renewal of “OTS964-suffered/survived” GS populations.

### Recovery in the size of cell population from OTS964-survived GS clones

**Figure 4:**
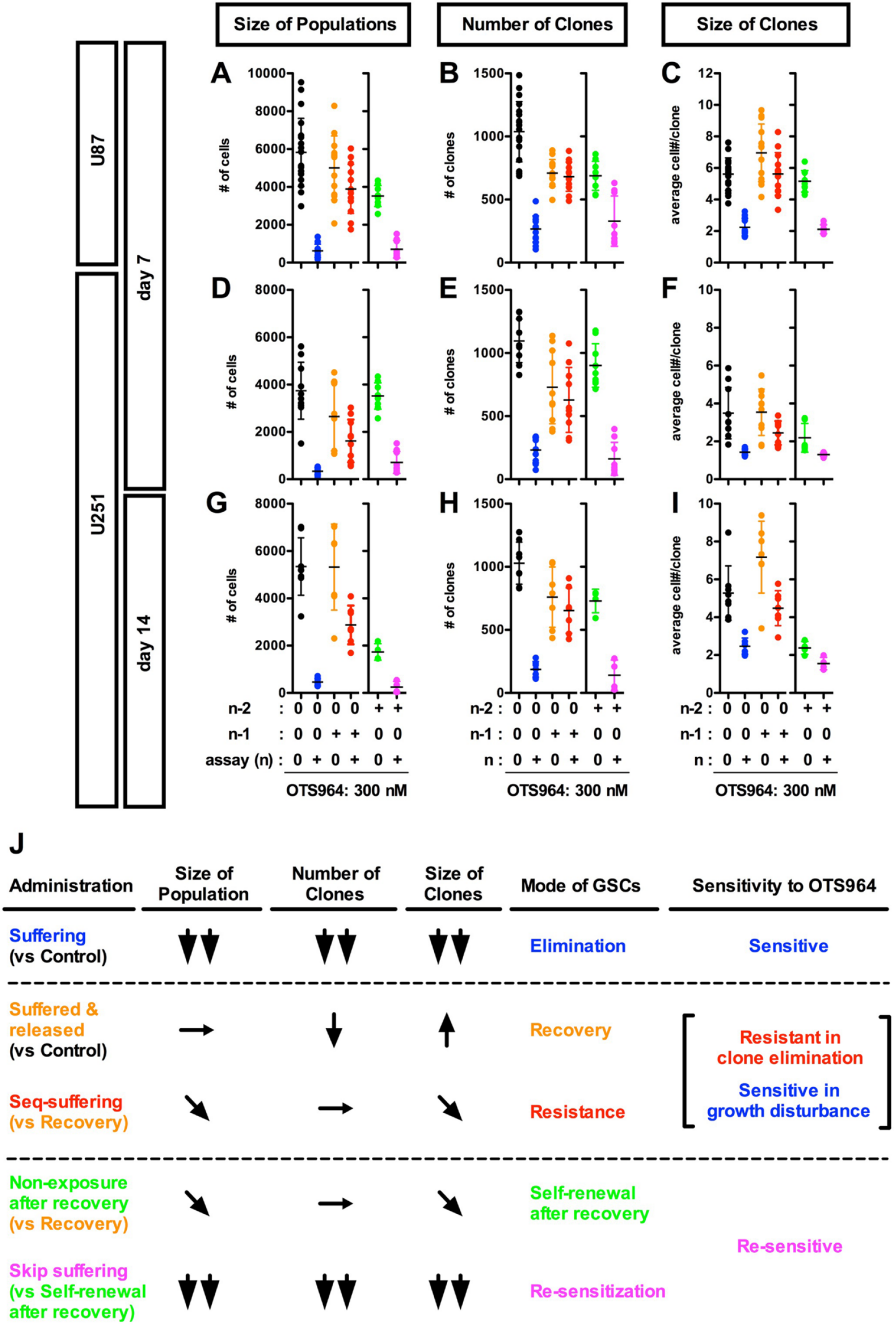
Recovery growth of U87- and U251-derived GS resistant clones after an exposure to and removal of OTS964; re-acquisition of the sensitivity to OTS964 in the recovered-GS populations during the self-renewal (**A**–**I**) U87- and U251-derived (A-C for U87-derived and D-I for U251-derived GS clones, respectively) GS clones were developed in the absence (0 nM, black circles for the non-suffering/non-suffered GS populations) and the presence of 300 nM (blue circles for the suffering/survived GS populations) of OTS964 in the assayed culture (*n*) at day 7 (A–F) and at day 14 (G–I). When 300 nM of OTS964 was administered in a generation (*n*–1), then an assay was conducted in the absence (orange circles for the released/recovered GS populations) and the presence (red circles for the seq-suffering/resisted GS populations) of OTS964 in the following assayed generations (*n*). When 300 nM of OTS964 was once administered at a generation (*n*–2) of GS clones, and then a population was recovered (*n*–1) in the absence of OTS964. The recovered population was again dissociated and assayed at the following generations (*n*) in the absence or presence (green for the self-renewed from the recovered, and magenta for the skip-suffering/re-sensitized GS populations, respectively) of OTS964. (**J**) A summary table for the phenotypes (in the size of populations, the number of clones and the size of clones); the mode of GSCs; the sensitivity to OTS964 in the different administration paradigms of OTS964.

We next asked whether OTS964-survived GS clones could recover the size of the original GS cell populations. We administered OTS964 to GS populations at a generation (*n*-1), and then collected OTS964-survived clones and passaged for the following assaying generations (@ n for an assay) (Supplementary Figure 3A and 3B). We found that both U87- and U251-derived OTS964-survived clones recovered the size of the GS populations at every following generation (Figure 4A, 4D, 4G and 4J; Supplementary Figures 3A, 3B, 5A, 5D, 5G and 6; see black, blue and orange labels). However, the number of “OTS964-recovered” clones was reduced compared to the non-suffered GS populations (Figure 4B, 4E, 4H and 4J; Supplementary Figures 3A, 3B, 5B, 5E, 5H and 6); on the other hand, we found an increasing tendency in the size of clones in both U87- and U251-derived OTS964-recovered GS populations (Figures 4C, 4F, 4I and 4J; Supplementary Figures 3A, 3B, 5C, 5F, 5I and 6). Thus, OTS964-survived GS clones recovered the size of the population which consisted of GS clones with less survival probability and with increasing growth tendency.

### OTS964-survived GS clones resist the clone-eliminating efficacy while allowing the growth-disturbing efficacy in the sequential exposure of OTS964

We next asked whether the OTS964-survived GS populations had become resistant to OTS964 by a sequential exposure paradigm (Supplementary Figure 3A and 3B). We administered OTS964 during the recovery growth of OTS964-survived GS populations, then, assayed the growth properties of the “sequentially re-suffering (seq-suffering)” GS populations. We found that seq-suffering GS populations did not reduce their number of clones compared to OTS964-released/recovered GS populations (Figure 4B, 4E, 4H and 4J; Supplementary Figures 3A, 3B, 5B, 5E, 5H and 6; see orange and red labels). Moreover, the number of seq-suffering GS clones was significantly higher than that of the first-suffering GS populations (Figure 4B, 4E, 4H and 4J; Supplementary Figures 5B, 5E, 5H and 6; see blue and red labels), suggesting that the OTS964-survived GS clones strongly resist the clone-eliminating efficacy of OTS964, demonstrating a resistant phenotype in the survival of GS clones.

As OTS964-survived GS clones showed a resistant phenotype to the clone-eliminating efficacy, the population size of the seq-suffering U87-derived GS populations was almost similar to the OTS964-released GS populations, suggesting that the U87-derived GS populations appeared to resist the size-reducing efficacy (Figure 4A and 4J; Supplementary Figure 3B, 5A and 6; see orange and red labels). Moreover, the growth-disturbing efficacy was significantly weaker to the seq-suffering GS clones than for the first-suffering GS clones in both the U87- and U251 derived GS populations, appearing that the seq-suffering GS clones resisted the growth-disturbing efficacy of OTS964 during the growth recovery (Figure 4C, 4F,4I and 4J; Supplementary Figures 2B, 2C, 3A, 3B, 5C, 5F, 5I and 6; see blue and red labels). On the other hand, the population size of the seq-suffering U251-derived GS populations was significantly smaller than the OTS964-released GS populations at day 14 (Figure 4G and 4J; Supplementary Figures 3B, 5G and 6; see orange and red labels). Consistently, the clone size of the seq-suffering U251-derived GS populations was significantly smaller than the OTS964-released GS populations (Figure 4I and 4J; Supplementary Figure 3B, 5I and 6); the clone size of multi-cellular clones in both U87- and U251-derived GS populations were significantly smaller that the OTS 964-released GS populations (Supplementary Figure 5C, 5F and 5I; Supplementary Figures 3B and 6; see orange and red labels). These showed that the sequential administration of OTS964 was less effective than that of the first administration, while the growth of the OTS964-survived GS clones had been significantly disturbed in the sequential administration. Thus, OTS964-survived GS clones reconstructed GS populations in the sequential exposure of OTS964 while resisting the clone-eliminating efficacy and allowing the growth-disturbing efficacy: these are referred to as “OTS964-resisted” GS populations (Supplementary Figure 3C).

### Plasticity in sensitivity/resistance to OTS964 during the self-renewal of OTS964-recovered GS populations

As described above, we have shown that OTS964-survived GS populations resist the sequential exposure during the recovery of growth suggesting that the OTS964-recovered GS populations have acquired resistant properties to OTS964. We thus hypothesized that the OTS964-recovered GS populations maintains the resistance over generations in self-renewal.

### Self-renewal of OTS964-recovered GS populations

We then asked whether OTS964-recovered populations could reproduce heterogeneous GS populations (Supplementary Figure 4A and 4B; see green boxes). We passaged the OTS964-recovered GS populations, and found that the number of surviving clones in the reproduced GS populations was similar to the pre-passaged ones (Figure 4B, 4E, 4H and 4J; Supplementary Figures 4A, 4B, 5B, 5E, 5H and 6; see orange and green labels). However, the growth of clones was significantly down-regulated in the reproduced GS populations (Figure 4C, 4F, 4I and 4J; Supplementary Figures 4A, 4B, 5C, 5F, 5I and 6), resulting in a significant reduction in the size of the reproduced GS populations (Figure 4A, 4D, 4G and 4J; Supplementary Figures 4A, 4B, 5A, 5D, 5G and 6). This suggests that OTS964-recovered GS clones were able to reproduce GS populations, while the growth of clones appeared to be down-regulated. Thus, OTS964-recovered GS populations self-renewed, demonstrating self-renewal of OTS964-recovered GS populations.

### Re-acquisition of sensitivity to OTS964 during the self-renewal of OTS964-recovered GS populations

We next asked whether GS clones derived from the OTS964-recovered GS populations resist the second exposure of OTS964 (skip-exposure; Supplementary Figure 4A and 4B; see magenta boxes) during their self-renewal. Surprisingly, we found that self-renewed GS clones derived from OTS964-recovered GS populations were re-sensitive to OTS964 in the skip-exposure experiments (Figure 4; Supplementary Figures 4A, 4B, 5 and 6; see green and magenta labels). The size of the GS populations remarkably shrunk due to significant reduction in both the number of clones and the growth of clones (Supplementary Figure 4C). This suggests that the GS clones from the OTS964-recovered GS populations had become re-sensitive to OTS964 during the self-renewal after the recovery.

### Robustness of power-law growth to OTS964 in GS populations

**Figure 5:**
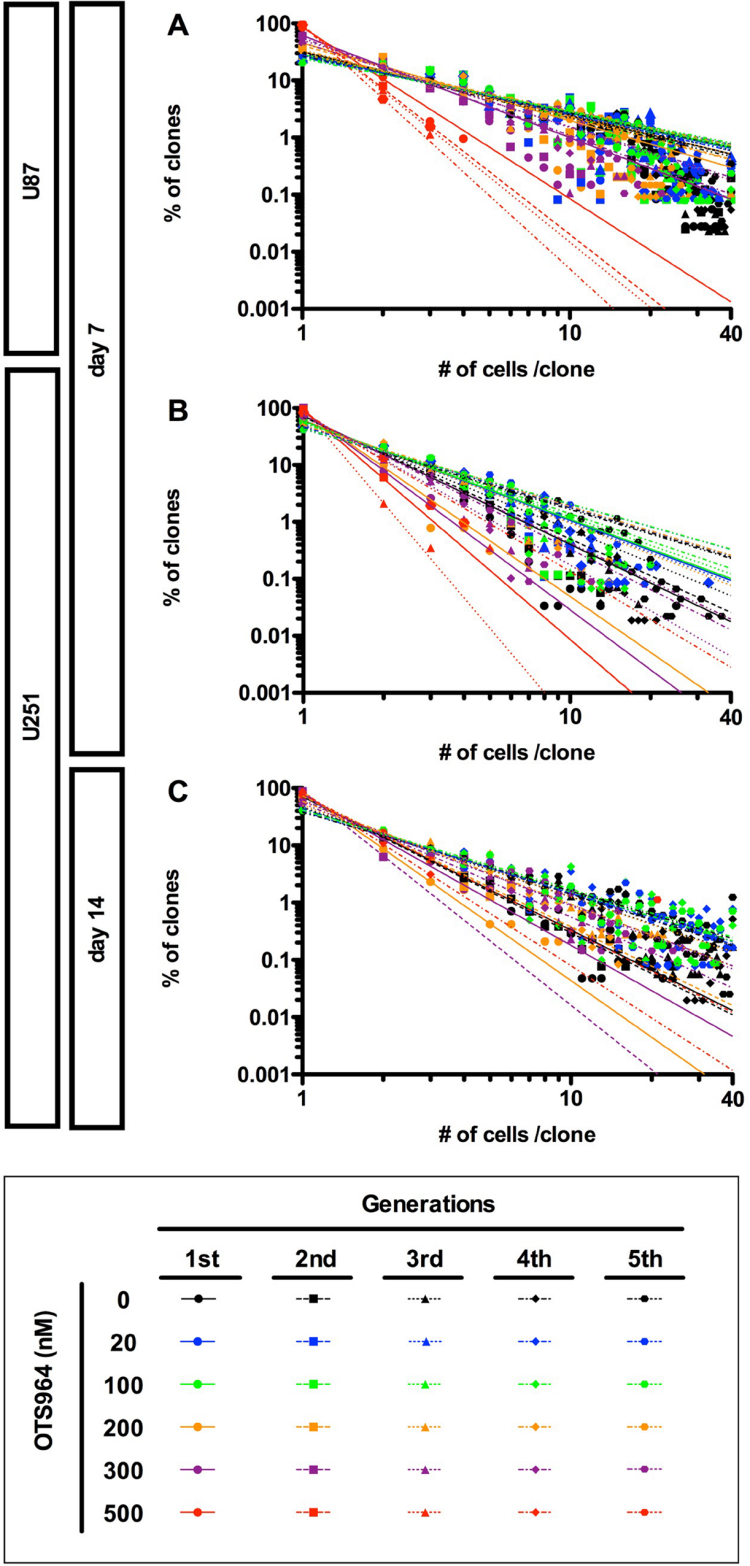
Recapitulation of diversity and power-law in growth during repeated passages in the presence of OTS964 (**A**–**C**) The graphs show double logarithmic plots of the clone size (number of cells in each clone) and the frequency of the GS clones at day 7 for the U87- (A) and the U251- (B) derived cell populations; at day 14 for the U251-derived cell populations (C). The all graphs showed that a power-law was recapitulated during repeated passages at different concentrations of OTS964. Thus, power-law in growth was maintained in the self-renewal of GS clones and of the GS populations even in the presence of OTS964.

In the previous study, we have discovered that the growth of GS clones follows a power-law, and that the power-law was not disrupted by the administration of an anti-glioma drug, TMZ, suggesting a robustness of power-law coded heterogeneous GS populations to an anti-tumor drug. We then asked whether the power-law growth was maintained in the presence of OTS964 in GS populations. We found that both the U87- and U251-derived GS populations repeatedly exhibited power-law growth at every generation in the presence of every different concentrations of OTS964 (Figure 5). Thus, power-law growth was maintained even in the OTS964-suffered/survived GS populations.

**Figure 6:**
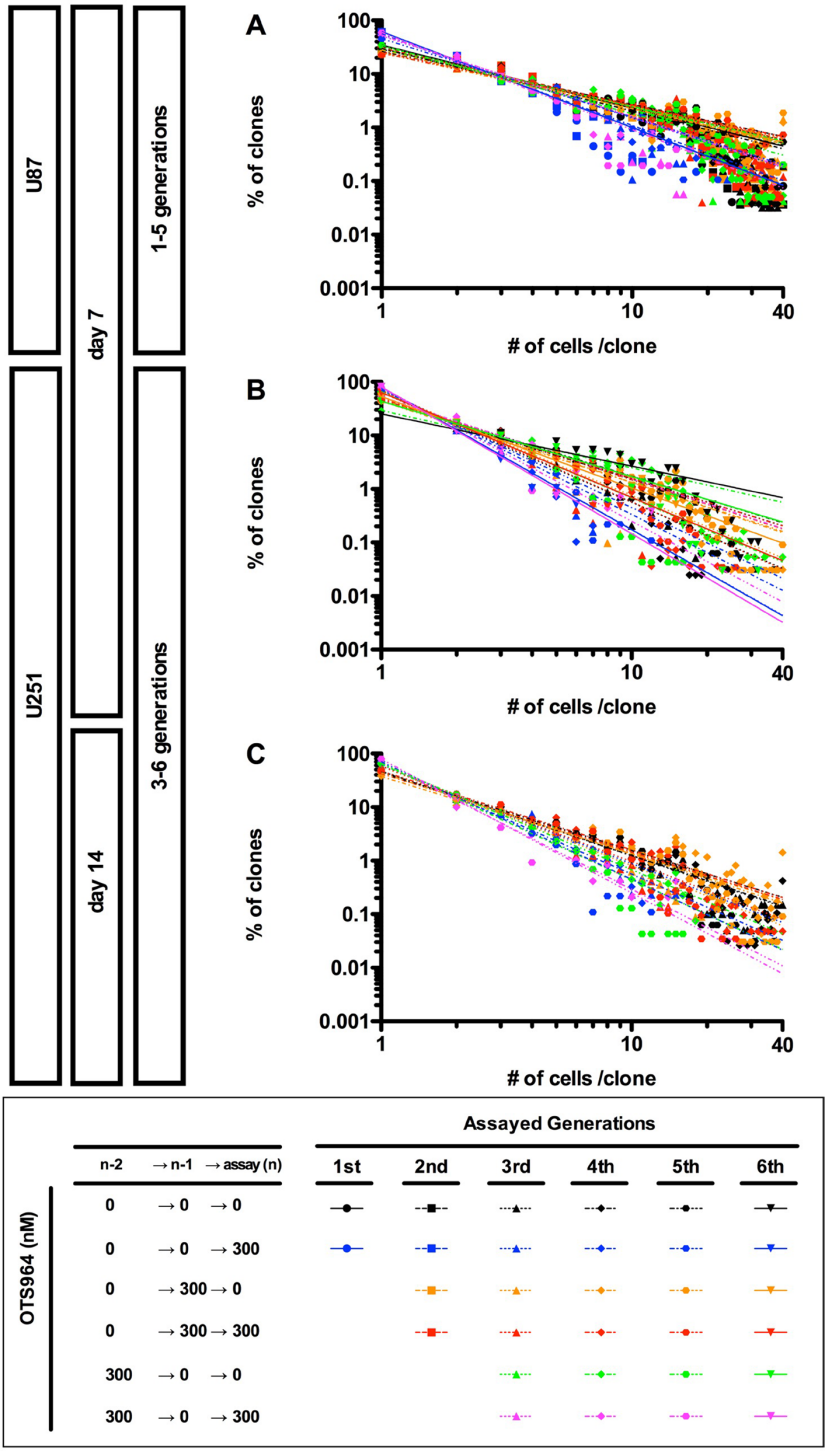
Recapitulation of diversity and power-law in growth during repeated passages in the sensitivity and the resistance to OTS964 (**A**–**C**) The graphs show double logarithmic plots of the clone size (number of cells in each clone) and the frequency of the GS clones at day 7 for the U87- (A) and the U251- (B) derived cell populations; at day 14 for the U251-derived cell populations (C). The all graphs showed that a power-law was recapitulated during repeated passages at different generations in every OTS964-administration paradigm. Thus, power-law growth was maintained in the self-renewal of functional heterogeneity to OTS964 in the GS populations.

Previously, we found that U87-GS populations recover the growth of clones while maintaining a power-law in the following generations after an administration of TMZ [9]. We then asked whether OTS964-recovered GS populations follow a power-law in growth (Supplementary Figure 3B). We found that both U87- and U251-derived OTS964-recovered GS populations recapitulated a power-law growth at every following generation after the exposure to OTS964 (Figure 6 and Supplementary Figure 3B; see orange labels). Thus, power-law coded GS populations were restored in the recovery growth from OTS964 exposure. We then found that the sequential exposure of OTS964 did not disrupt a power-law growth suggesting that OTS964-resisted GS populations follow a power-law. Thus, a power-law was never disrupted during the recovery growth of the OTS964-survived clones (Figure 6; see red labels). We further found that the OTS964-recovered GS clones recapitulated a power-law during the self-renewal (Figure 6; see green labels). Thus, the power-law coded OTS964-recovered GS populations self-renewed.

We finally asked whether re-sensitization to OTS964 is associated with a disruption of power-law in the skip-exposure. The OTS964-re-sensitized GS populations again exhibited a power-law growth as well as previously sensitized populations (Figure 6; see magenta labels). Thus, a power-law growth maintains the heterogeneous GS populations while suffering from size reducing efficacies of OTS964, suggesting robustness of power-law coded heterogeneous GSC populations.

## DISCUSSION

### OTS964 reduces the size of heterogeneous glioma stem cell populations

Tumor size is a most important issue for brain tumors, because the volume in the skull is limited, and thus the progression of an intracranial brain tumor occupies the limited space and causes herniation of other parts of the brain [9, 30]. Thus, any treatment for brain tumors would tightly be linked with controlling size of the tumors [9]. The tumor neurosphere system enables us to quantitatively identify the size of GSC populations and the functional properties such as the survival, the growth, the self-renewal and plasticity. This kind of quantitative system would be necessary for establishing a novel “size-controlling” chemotherapy in which CSCs are targeted to be dysfunctional [2, 7, 9, 27, 28].

This study showed that OTS964 reduced the size of GS populations via distinct paths. First, OTS964 eliminated the GS clones in a dose dependent manner, showing that OTS964 directly kills GSCs via shutting-down the kinase pathway essential for the cell survival. This efficacy would cause shrinkage in the size of GSC populations after the drug administration. Second, OTS964 also disturbed the growth of GS clones in a dose dependent manner. This disturbing efficacy was further observed over the long-term after the released from OTS964 over generations. The biphasic disturbances suggest that OTS964 down-regulates the proliferation of GSCs not only via a short-term signaling inhibition but also via possible long-term epigenetic effects [18]. Thus, OTS964 shrinks the size of GSC populations via suppressing the survival and the growth of GSC clones, possibly suggesting a mechanism for long-term control of recurrence of glioblastoma [31–33].

The survival and the growth of the U87- and U251-derived GS populations appeared to be different showing that the survival and the growth can be dependent on the types of cells. This suggests that patient-derived GSCs may exhibit diverse properties in the growth of the GSC populations. Moreover, the facts that GS clones/cells enhanced the survival and the growth in this study compared to the previous suggest that the GSCs can plastically change the state of the growth properties [9]. The diversity and the plasticity may be able to affect the efficacy of OTS964. Thus, it is further needed to address the efficacy of OTS964 by using patient-derived GSCs.

### Distinct mode of OTS964-sensitive and OTS964-resistant GS clones in the survival and the growth

We showed that OTS964-suffered GS clones recovered the population size in every following generation. The recovery growth showed less survival probability and more growth tendency. The changes of the phenotypes in OTS964-recovered GS clones reflect changes in properties in the survival and the growth from the non-suffered GS clones. Then, OTS964-suffered GS clones were sequentially re-suffered from the OTS964, the “seq-suffering” GS clones showed a consistent probability in survival and a reduction in clones size suggesting that the “seq-suffering” GS clones resist the clone-eliminating efficacy while allowing the growth-disturbing efficacy. This again suggests that the OTS964-suffererd GS clones have changed the properties in their survival and growth from an “OTS964-senstive mode” to a “resistant mode”.

The OTS964-suffered/recovering GS clones appear to have been “selected” by OTS964. The facts that the “selected” GS clones exhibit less survival probability and show resistance to OTS964 suggest that survival-supporting mechanisms for an “OTS964-sensitive mode” and for an “OTS964-resistant mode” are not qualitatively similar but rather distinct. Alternatively, a survival supporting mechanism in an “OTS964-sensitive mode” appears to have plastically been replaced by qualitatively distinct “OTS964-resistant mode” during the selection.

On the other hand, the “selected” GS clones grew more rapidly in the recovery growth; the “selected” GS clones were again disturbed the growth by OTS964. This suggests that a mechanism for the growth of GS clones in an “OTS964-sensitive mode” is quantitatively enhanced in the “selected” GS clones. That is, the growth for an “OTS964-resistant mode” is an exaggerated “OTS964-sensitive mode”. Thus, OTS964-suffered GS clones have been made resistant by developing a qualitatively distinct mechanism for survival and enhanced growth during the “selection”.

### Functional heterogeneity and plasticity of glioma stem cell populations in sensitivity/resistance to OTS964

The major problem to be conquered in cancer chemotherapies is resistance of tumors to anti-tumor agents [5]. Mechanistically, how can cancer cells be resistant, or practically how does cancer appear to be resistant [5, 6, 34]? The situation, clinically, is that a “remaining” tumor after chemotherapy “reinitiates” growth, then this re-growth of the tumor appears to have become “resistant” to the chemotherapy. Microscopically, cells may down-regulate an importing system for the agent or may up-regulate an exporting or degrading system. Macroscopically, such “resistant” cells may survive, and may re-grow dominantly [31, 34, 35]. Then, the re-growing tumor may appear to be irreversibly resistant to the chemotherapy. However, functional heterogeneity in tumor initiating cells/CSCs raises another issue, specifically whether heterogeneous CSC populations show heterogeneity in “resistance”. If so, the questions are whether the resistance is irreversibly cemented or reversibly plastic. Both cases can result in heterogeneity in resistance in CSC populations. If the former is the case, how do qualitatively and quantitatively different resistant types reside in a CSC population? If the latter, how quantitatively plastic CSCs become resistant would be the matter. Thus, we should focus on the “resistance” and its “heterogeneity” to be targeted in both qualitative and quantitative manner.

We previously showed a functional heterogeneity in GSC populations where cells/clones heterogeneously reproduce another cell/clone in a power-law coding probabilistic manner, suggesting that the intra-/inter-clonal heterogeneity was not qualitatively differentiated but plastically changed in GSC populations [9]. This study showed another functional heterogeneity, sensitivity/resistance to OTS964: where OTS964-surivived GS clones resisted the sequential exposure during the recovery of growth; where the OTS964-recovered GS populations did not sustain the resistant properties during the self-renewal. If GS clones do not own intra-clonal heterogeneity, a GS population consisted of qualitatively differentiated sensitive-only (S-only) and resistant-only (R-only) clones in a mutual exclusive manner; S-only clones would just be eliminated in a dose dependent manner. Then, the passage of OTS964-survived clones would cause an enrichment of R-only clones. On the other hand, OTS964-survived/resistant clones may be intra-clonally heterogeneous where clones consist of both sensitive and resistant cells (S&R-clones). Then, an OTS964-survived/resistant population may consist of R-only and/or S&R clones. If all clones consisted of R-only and/or S&R clones, clones should have survived with no decrease in the number of survived clones in the presence of OTS964. The fact that significant reduction in the number of GS clones in the presence of OTS964 suggests that there must be some S-only clones in the presence of GS populations. Even if GS populations consisted of irreversibly differentiated S-only, R-only and S&R clones, the resistant clones should be getting enriched in the following generations. However, our results where resistant clones never irreversibly enriched did not support either case, rather suggest that OTS964-exposed clones reversibly acquire resistant properties to be R-only or S&R-clones. That is, the functional heterogeneity in a chemo-resistance appears to be stochastically changed. Thus, heterogeneity in growth and chemo-sensitivity/resistance were not the consequence of irreversible differentiation, nor the clonal expansion of resistant cells/clones. It rather suggests that the heterogeneity was a transitional phenotype of cellular/clonal “states”, and that cells/clones are reproducibly plastic. And, thus, the GSC populations continuously exhibit heterogeneity, and sustain themselves from an anti-tumor agent by taking advantage of the stochastic plasticity [36].

As discussed above, the plasticity can explain re-acquisition of sensitivity to OTS964. However, the GSC clones may need some time to re-acquire sensitive properties, as shown in experiments where OTS964 was administered in sequential generations vs skipped generations: while sequential-exposure resulted in a consistent survival of clones, skip-exposure resulted in a significant reduction in the survival of clones, respectively. In other words, the continuous exposure of OTS964 to GSC populations may stimulate and sustain a mechanism to acquire resistant properties suggesting “washing-drugs-out” may down-regulate a possible sustaining mechanism leading an re-acquisition of drug sensitivity for GSCs [31].

### Robustness of OTS964-sensitive heterogeneous glioma stem cell populations via maintaining a power-law

We showed that every OTS964-sensitive GS populations followed a power-law in the presence of various drug concentrations. We further showed that OTS964-survived, -recovered, -resisted and re-sensitized GS populations never disrupted a power-law, suggesting that the power-law coding supports spatial self-similarities in the sensitivity/resistance to OTS964 in the heterogeneous GSC populations. Moreover, the GS populations reproduced over generations and always followed a power-law, suggesting that the spatial self-similarities are also temporally preserved.

We previously proposed a hypothesis in which disruption of the power-law controlling mechanism might be able to destroy reproducibility of heterogeneous GSC populations [9]. OTS964 did not destroy a power-law growth and plasticity of the GS populations, suggesting that OTS964-surivived population would be self-renewable with following a power-law. These also suggest that TOPK is not responsible for the power-law growth in the heterogeneous GSC populations. Thus, GSC populations are spatially and temporally reproduced via maintaining a power-law during the self-renewal [9, 37, 38], that is, robustness of functionally heterogeneous GSC populations.

### A therapeutic strategy to a malignant brain tumor by OTS964

Even though the combination of radiation and TMZ has been a standard therapy to glioblastoma, patients still present with a poor prognosis [39]. In the previous study, we showed that TMZ disturbed the growth of U87-derived GSC populations, while significant maintenance of clones [5, 9]. In this study, we showed that OTS964 disturbed growth and survival of GS clones resulting in significant shrinkage of GS populations. The major difference between TMZ- and OTS964-suffered populations is the survival rate of GS clones suggesting that OTS964 has stronger clone-eliminating efficacy to GSC populations. Moreover, survival rates of the recovered GSC populations from the TMZ-suffered populations appear to be higher than the OTS964-recovered populations, again suggests that OTS964 is superior to TMZ in the clone-eliminating efficacy. On the other hand, the growth recovery of the TMZ-suffered GSC clones appears to be more restricted than of the OTS964-survived clones, suggesting that TMZ is superior to OTS964 in controlling the growth recovery of drug-suffered clones for a long-term. The efficacies of TMZ and OTS964 appear to be complimentary so that a combination treatment would be able to help to shrink the size of GS populations for over generations. Then, it would take more generations for the recovery of the initial size of GSC population connecting to a control of recurrence of glioblastoma for a long term. However, both of them never disrupted “power-law growth”, so that we speculate that a “TMZ&OTS964-suffered GSC population” may be able to sustain self-renewal activity connecting to future recurrence after a long-term. Thus, a combined therapy with TMZ and OTS964 may benefit patients in a long-term control of recurrence by regulating the size/survival/growth of the GSC populations [5, 9, 40], while the “TMZ&OTS964-suffered GSC population” may sustain self-renewal leading to slow recurrence of glioblastoma.

Another major issue is plasticity of GSCs. Radiation gives an irreversible damage to the chromosomes resulting in a possible restriction of plasticity to GSCs [39]. Then, radiation therapy would promote irreversible impairment in reconstruction of GSC populations via restricting the plasticity, however, the efficacy would be dependent of radiation doses associated with increasing side effects [41]. Thus, we need another help to control the plasticity of the resistant/remaining GSCs while decreasing radiation doses [41]. We showed that the OTS964-recovered GSCs plastically down-regulated resistant activity, then re-acquire sensitivity to OTS964 in the skip-exposure. This suggests that the sustained reversible plasticity of GSCs in the drug sensitivity can beneficially promote a repeated elimination of the GSC clones, and that repeated short-term administration paradigm which mimics the skip-exposure of OTS964 may repeatedly be able to suppress the growth. By taking advantage of the sustained reversible plasticity of GSCs after OTS964 administration, you may also be able to reduce the radiation doses [39, 41]. Thus, a combined therapy with the current standard and OTS964 would benefit patients in a long-term control in recurrence of glioblastoma via a repeated size reduction of GSC populations.

## MATERIALS AND METHODS

### Clonal assay and repopulation experiments

The cell lines U87 (RCB419, RIKEN BRC, Tsukuba, Japan; HTB-14, ATCC) and U251 (RCB0461, RIKEN BRC) were selected for study because of their sphere-forming ability and because these lines significantly expressed TOPK (Hayashi *et al.*, in press; Supplementary Figure 1A and 1B). To develop U87- and U251-derived tumor neurospheres/glioma spheres (GSs), single glioma cell line-derived cells were cultured under non-adherent conditions using poly(2-hydroxyethyl methacrylate) (polyHEMA, Sigma-Aldrich, St. Louis, MO, USA) coated 60-mm dishes (BD Biosciences, Franklin Lakes, NJ, USA) (400,000 cells/dish). The GS clones survived and grew to become GSs, then the GSs were dissociated to passage for following generations (Supplementary Figure 2A). To determine the frequency (i.e., the proportion) of self-renewing GSs in the population, cells were dissociated with 0.1% trypsin + 400 µM EDTA (ThermoFisher SCIENTIFIC, Carlsbad, CA, USA) and seeded in growth medium containing 0.8% methylcellulose (Nacalai Tesque, Kyoto, Japan) on day 0 in poly-HEMA coated 12-well plates (BD Biosciences) at a clonal density of 4,000 cells/2 ml/well, then the GSs were measured and the size of GS clones was quantified by counting the number of cells in the 1/2 well area [9]. Even if the clones survive as single-cell or less-numbered clones, we considered them “GS clones” in this study [9]. Number of clones and number of cells/clone were quantified at days 4, 7, 14, 21 and 28. For details see previous studies [9, 27–29, 42]. GS-containing populations were passaged five to six times. Cells derived from each passage were subjected to clonal assays. Each series of repopulation experiments were repeated four times [9].

OTS964 (kindly provided by OncoTherapy Science, Inc., Kawasaki, Japan) was dissolved in DMSO. We, first, prepared 100 times of various concentrations of OTS964, and then administered them to culture media in which the final concentration of DMSO to be 1%. The control experiment contains 1% of DMSO, too.

### Assay for “clone-eliminating” and “growth-disturbing” efficacies of OTS964 to GS Clones in the heterogeneous GS populations

OTS964 was administered at 20, 100, 200, 300 and 500 nM in the assaying culture, then the number of cells in each clone quantified in both the presence and the absence (0 nM) of OTS964 (@ n for assay; Supplementary Figure 2B). We defined 300 nM as the concentration with which we conducted further experiments. Because 300 nM of OTS964 significantly decreased the number of both U87- and U251-derived GS clones to about less than 1/4, however, we still were able to re-collect enough of the remaining surviving GS clones for the following experiments. We subsequently use the “OTS964-survived” GS populations for the survived GS populations after suffering from OTS964.

If “OTS964-suffering” GS clones were eliminated, there should be a decrease in the number of clones indicating less survival of clones. If the growth of OTS964-suffering GS clones was disturbed via suppression to the self-renewal and/or to the clonal expansion, there should be a decrease in the number of cells in a clone due to less growth of clones. Thus, we can identify “clone-eliminating” and “growth-disturbing” efficacies of OTS964 to GS clones in the tumor neurosphere system [9]. The series of experiments were repeated four times (from 3rd to 5th generations for U87; 4th to 6th for U251 in each series).

### Assay for “recovery” and “resistance” of GS clones after administration of OTS964

U87- and U251-derived GS clones were treated with OTS964 (@ (*n*–1)), and then the “OTS964-survived” GS clones were passaged/dissociated to following generations (@ *n* for assay) (Supplementary Figure 3A). The dissociated GS clones were allowed to grow in the absence of OTS964: namely, the GS clones had been “released” (This is referred to as “OTS964-released” GS clones). Thus, we could address whether the “OTS964-survived/released” GS populations recover the population size [9]. By contrast, the survived GS clones were allowed to grow in the presence of OTS964 again: namely, the GS clones were sequentially designated as “re-suffering” from OTS964 (This is referred to as “seq-suffering” GS clones) for whether the “OTS964-survived/seq-suffering” GS clones exhibit resistance to sequential exposure of OTS964 during the growth recovery (Supplementary Figure 3A and 3B). The series of experiments were repeated four times (2nd to 5th generations for U87; 4th to 6th for U251 in each series, respectively). We subsequently use the “OTS964-recovered” GS populations for the GS populations after recovering from OTS964.

### Assay for “re-acquisition of sensitivity: re-sensitivity” to OTS964 in the “self-renewal” of the recovered GS populations

U87- and U251-derived GS clones suffered from OTS964 (@ (*n*–2) generation), and then the OTS964-survived/released GS clones re-constructed/recovered GS populations (@ (*n*–1) generation). The OTS964-recovered populations were again passaged/dissociated to the following generations (@ *n* generations for assays), and then assayed in the absence of OTS964: namely, the passaged GS clones were “not suffering” from OTS964 for addressing whether the “OTS964-recovered” GS populations self-renew. By contrast, the OTS964-recovered populations were allowed to grow in the presence of OTS964: namely, the GS clones were intermittently re-suffering (This is referred to as “skip-suffering” GS clones) of OTS964 for addressing whether the “OTS964-recovered” GS populations maintain resistance to OTS964 during the self-renewal (Supplementary Figure 4A and 4B). The series of experiments were repeated three times (3rd to 5th for U87; 4th to 6th for U251) [9, 28].

### Semiquantitative reverse transcription-PCR analysis

We extracted total RNA from each of cell lines using RNeasy kit (Qiagen, Valencia, CA, USA). RT-PCR analysis was performed using OneStep RT-PCR kit (Qiagen) with gene-specific primers of GAPDH as internal control and TOPK; 5′-ATGGAAATCCCATCACCATCT-3′ and 5′-GGTTGAGCACAGGG-3′ for GAPDH and 5′-GCCTTCATCATCCAAACATT-3′ and 5′-GGCAAATATGTCTGCCTTGT-3′ for TOPK, which yielded a 1,000 and 425 bp product, respectively [18]. Amplified PCR products were analyzed on a 2% agarose gel and visualized with ethidium bromide staining.

### Western blot analysis

To detect the endogenous TOPK protein in glioma cells, cells were lysed in lysis buffer; 50 mmol/L Tris-HCl (pH 8.0) containing 150 mmol/L NaCl, 0.5% NP-40 and 0.1% protease inhibitor cocktail III (Calbiochem, San Diego, CA, USA). Cell lysates were prepared as described [18]. The amount of total protein was estimated by protein assay kit (Bio-Rad, Hercules, CA, USA), and then proteins were mixed with SDS sample buffer and boiled before loading at 10% SDS-PAGE gel. After electrophoresis, the proteins were blotted onto PVDF membrane (Merck Millipore, Billerica, MA, USA). Membranes, including proteins, were blocked by blocking solution and incubated with anti-PBK/TOPK monoclonal antibody (BD Biosciences, San Jose, CA, USA) for detection of endogenous TOPK protein. Finally, the membrane was incubated with horseradish peroxidase-conjugated secondary antibody and protein bands were visualized by enhanced chemiluminescence detection reagents (GE Healthcare, Buckinghamshire, UK). ß-Actin was examined to serve as a loading control.

### Determination of IC_50_ for OTS964

*In vitro* cell viability was measured by the colorimeteric assay using WST-1 reagents (Roche Diagnostics, Indianapolis, IN, USA). U87 and U251 cells (1 × 10^3^ cells/100 µl) were plated in 96-well plates. The cells were allowed to adhere overnight before exposure to various concentrations of OTS964 for 72 hours at 37°C. Plates were read with a spectrophotometer at a wavelength of 450 nm. All assays were carried out in triplicate. The values of IC_50_ were calculated by the straight line system made from 2 points of concentration of OTS964 around the 50% inhibition of cell viability.

### Graphs and statistical analyses

All graphing and regression analyses were done using GraphPad Prism version 5.0b for Mac OS X (GraphPad Software, Inc., San Diego California USA, www.graphpad.com.).

## SUPPLEMENTARY MATERIALS FIGURES


