# Supplementary material for: Targeting the T-Lak cell originated protein kinase by OTS964 shrinks the size of power-law coded heterogeneous glioma stem cell populations
Source: Oncotarget. 2017 Dec 9;9(3):3043–59. doi: 10.18632/oncotarget.23077 (PMC5790444; doi:10.18632/oncotarget.23077)
Supplement: Supplementary file 1 [file oncotarget-09-3043-s001.pdf]

Targeting the T-Lak cell originated protein kinase by OTS964 shrinks the size of power-law coded heterogeneous glioma stem cell populations

SUPPLEMENTARY MATERIALS

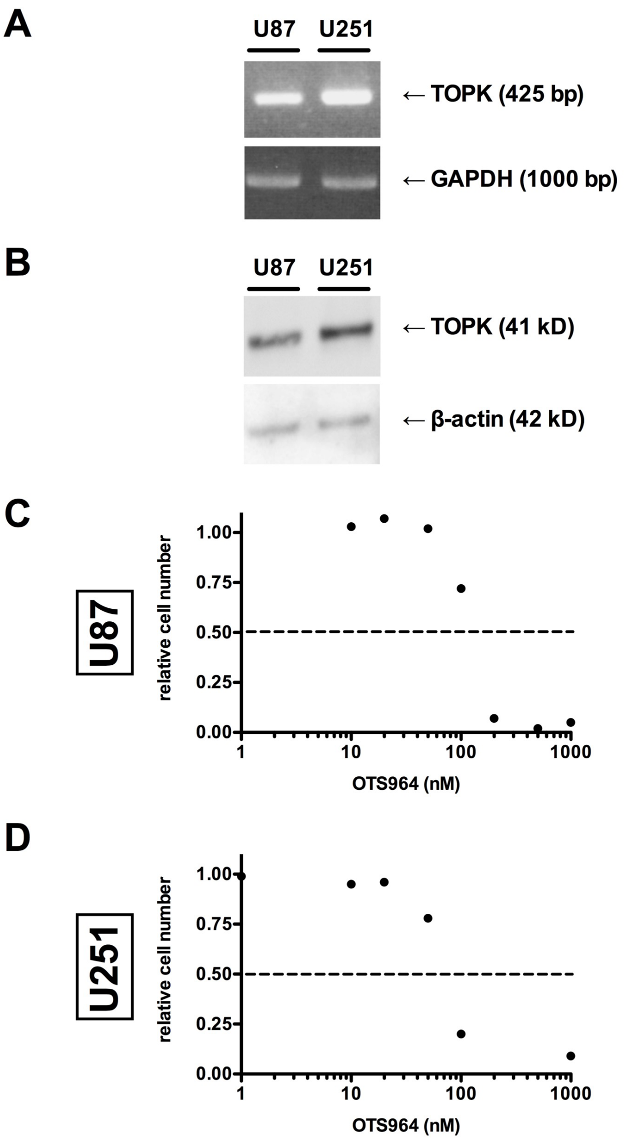

Supplementary Figure 1: Expression of TOPK in the U87 and U251 glioma cells; *in vitro* cell viability assay for U87 and U251 glioma cells in various concentrations of OTS964. (**A**, **B**), Expression of TOPK in U87 and U251 glioma cells in the semiquatitative RT-PCR analysis (A) and in the Western blot analysis (B), respectively. Expression of TOPK is more significant in the U251 than in the U87 glioma cells. (**C**, **D**) The graphs show the data of cell viability assay for U87 and U251 glioma cells in various concentrations of OTS964 (C and D for U87 and U251, respectively). The data allowed us to determine the IC_{50}s of U87 and U251 glioma cells as 125 nM and 68 nM, respectively.

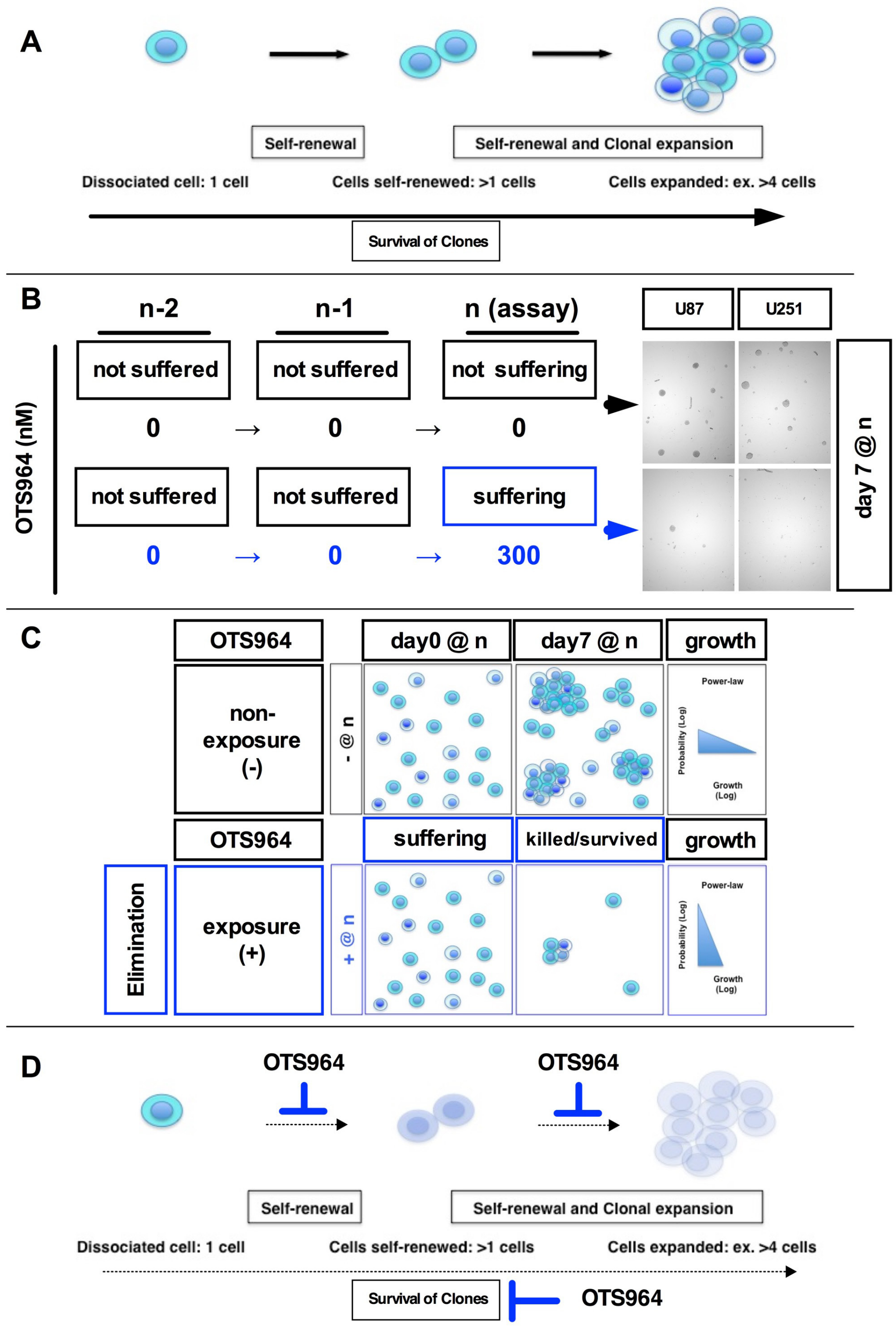

Supplementary Figure 2: OTS964 kills and disturbs the growth of GS clones in the tumor neurosphere culture. (**A**) A schematic diagram of the growth of GS clones where GS clones survive, self-renew and expand for maintaining themselves. (**B**) The experimental paradigm for assaying an efficacy in size control of heterogeneous GS populations (left): GS clones, were assayed in the absence/presence of 300 nM of OTS964 (see above, written in black letters for the absence; and below, in blue letters for the presence, respectively) at an assay (*n*-th for an assay) generation. Representative pictures of U87- and U251-derived GS clones in the tumor neursphere assay (right). (**C**) Size-reducing efficacy of OTS964 in GS populations: OTS964 killed GS clones, and disturbed the growth of suffering GS clones at the assay generations; while GS populations still maintained a power-law growth. The control (non-exposure: −) and the administration (exposure: +) experiments are in the black- and in the blue-lined boxes, respectively. (**D**) A schematic diagram of the efficacy of OTS964, where OTS964 suppresses the survival, the self-renewal and clonal expansion of GS clones.

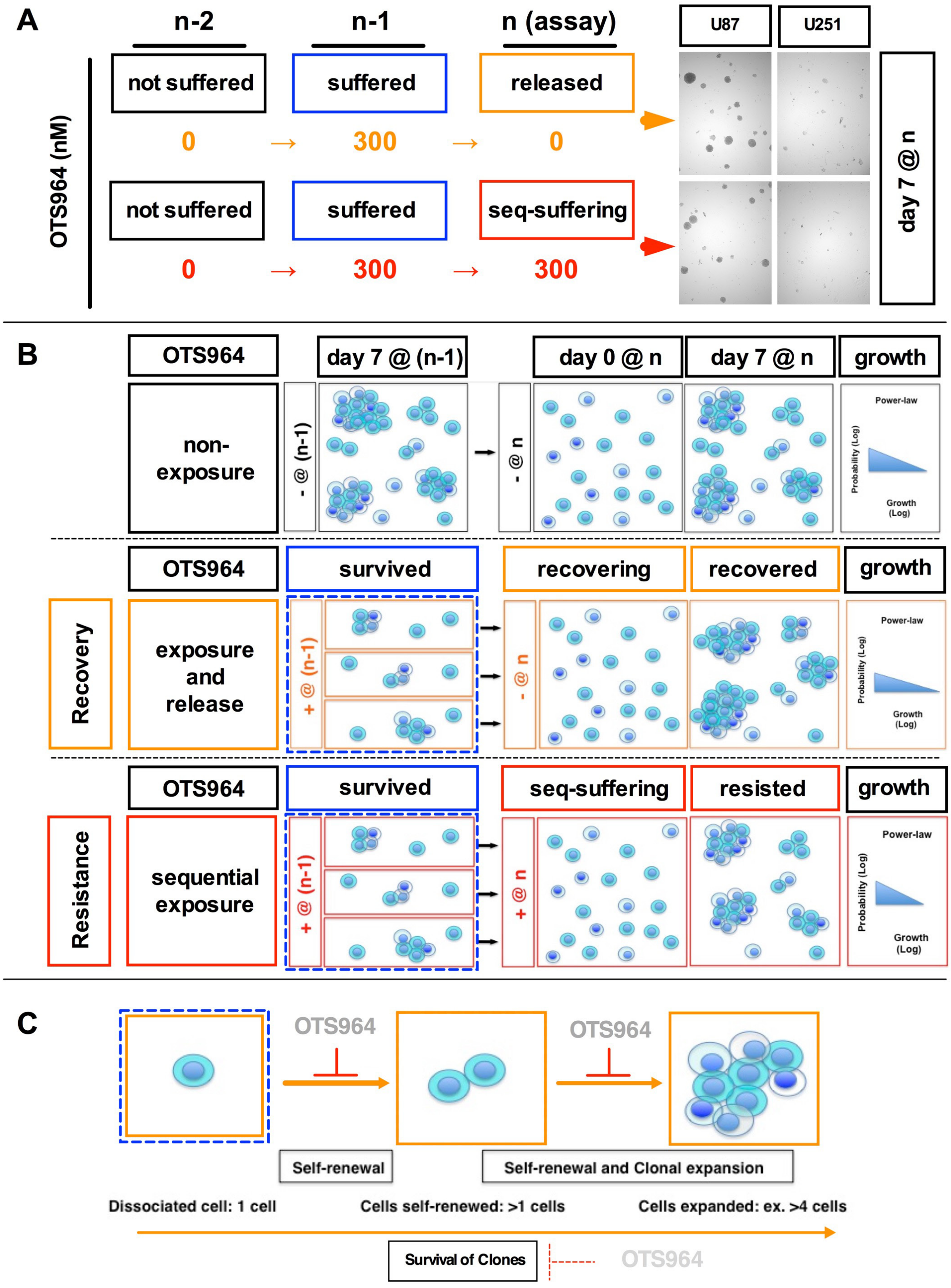

Supplementary Figure 3: Recovery and resistance of OTS964-suffered GS populations while maintaining a power-law. (**A**) The experimental paradigms for assaying the recovery and the resistance of heterogeneous GS populations after administration of OTS964 (left): GS clones, were suffered from 300 nM of OTS964 at (*n*–1)-th generations, and were conducted to assays. The released and the sequentially suffering (seq-suffering) GS clones (boxed in orange and red lines, respectively) were assayed at an assay (*n*: assay) generation. Representative pictures of the released/seq-suffering GS clones derived from U87- and U251-cell lines (right). (**B**) Schematic diagrams for series of the control-, the recovery- and the resistance-assaying experiments (boxed in the black-, orange- and red-lines, respectively). GS populations were suffered and survived to exposure of OTS964 (@ (*n*–1); boxed in the dashed blue-lines; they are in line with the survived populations shown in Supplementary Figure 2C), and then passaged/dissociated to conduct assays in the following generations (@ *n*). OTS964-suffered GS populations recovered the growth and the size of populations, while they exhibit resistance to the sequential exposure of OTS964 during the recovering phase. Power-law growth was maintained in the recovered and resisted GS populations. (**C**) A schematic diagram of the growth of OTS964-suffered GS clones. OTS964-sufferered GS clones (boxed in dashed blue-lines) survive, re-grow and expand for recovering the size of GS populations (Boxed/drawn in orange lines/arrows); resist the clone-eliminating efficacy in the sequential exposure of OTS964 during the recovering growth (indicated in the blunt ended dashed red-lines).

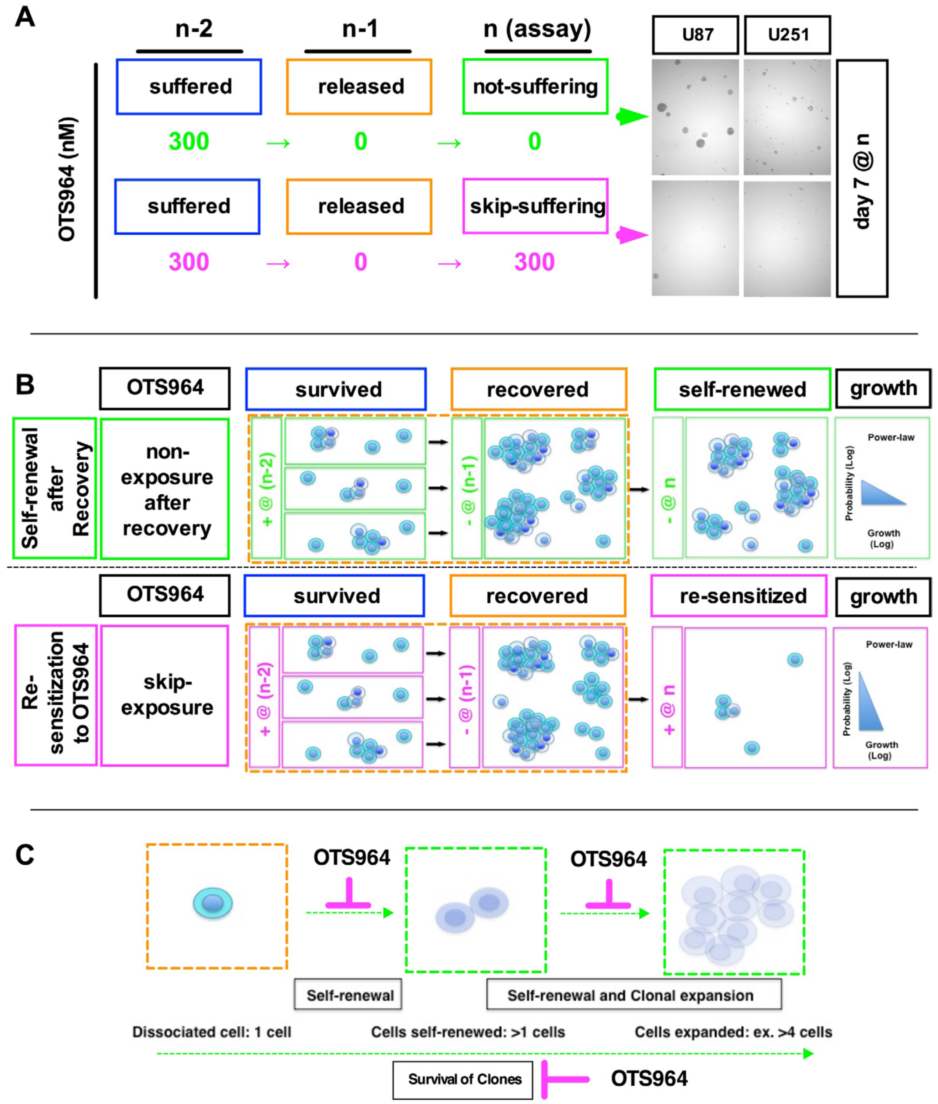

Supplementary Figure 4: Self-renewal of the recovered GS populations and re-sensitization to OTS964 while maintaining a power-law. (**A**) The experimental paradigm for assaying the sensitivity to OTS964 during the self-renewal of recovered heterogeneous GS populations (left): GS clones, were suffered from 300 nM of OTS964 at (*n*–2)-th generations (boxed in blue lines), then released from OTS964 to recover the size of the GS populations at (*n*–1)-th generations (boxed in orange lines). The recovered GS populations were dissociated to conduct assays to address if the recovered GS populations are able to self-renew in the following n-th generations (boxed in green lines). The *n*-th GS clones were grown and assayed in the absence/presence (not-suffering in the green-lined box for the absence, skip-suffering in the magenta-lined box for the presence, respectively) of OTS964 during the self-renewal of the recovered-GS populations. Representative pictures of the not-suffering/skip-suffering GS clones derived from U87- and U251-cell lines (right). (**B**) Schematic diagrams for series of experiments for assaying the self-renewal and for the re-sensitivity (boxed in the green- and magenta-lines, respectively). GS populations were suffered and survived to exposure of OTS964, and then passaged/dissociated to recover the size of GS populations (@ (*n*–2) and @ (*n*–1); boxed in the dashed orange-lines; they are in line with the diagrams shown in Supplementary Figure 3B). The recovered GS populations were passaged to conduct assays in the following generations (@ *n*) in the absence/presence of OTS964 (green- and magenta-lined boxes for the absence and the presence of OTS964, respectively). The recovered GS populations were able to self-renew (self-renewed: in the green-lined box), while they exhibit re-sensitivity to the skip-exposure of OTS964 during the self-renewal (re-sensitized: in the magenta-lined box). Power-law growth was maintained in both the self-renewed and re-sensitized GS populations. (**C**) A schematic diagram of the growth of the recovered GS clones during the self-renewal. OTS964-recovered GS clones (boxed in dashed orange-lines) survive, re-grow and expand for self-renewing the GS populations (boxed/drawn in dashed green lines/arrows); the recovered "once-resistant" GS clones re-sensitize to the skip-exposure of OTS964 (suppression of the survival, of the self-renewal and of the clonal expansion, indicated in the blunt ended magenta-lines).

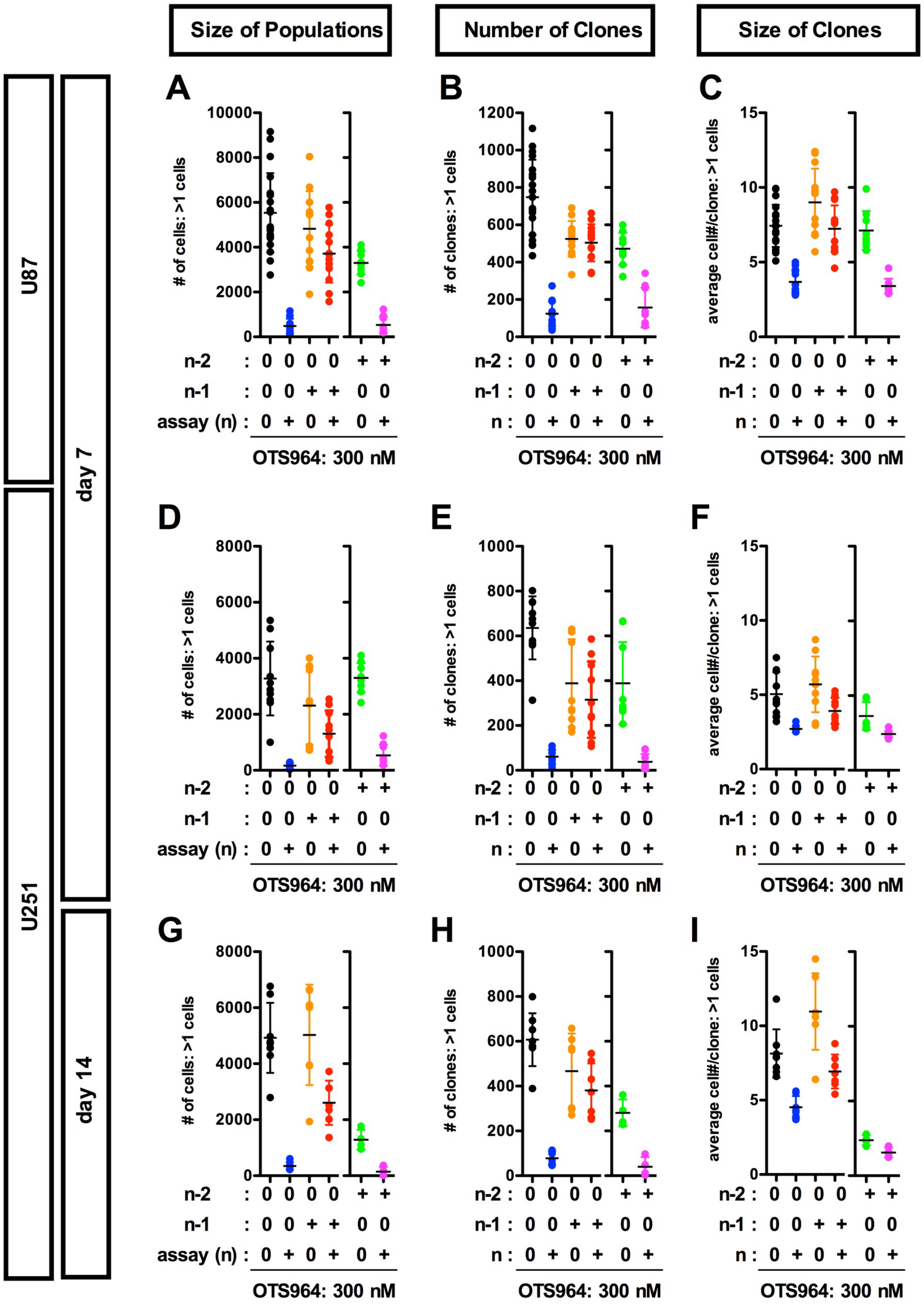

Supplementary Figure 5: Functional reproducibility of self-renewed GS clones in the sensitivity and the resistance to OTS964. (**A–I**) The data came from the self-renewed clones of U87- and U251-derived (A–C for U87-derived and D–I for U251-derived GS clones, respectively). GS clones were developed in the absence (0 nM, black circle) and the presence of 300 nM (blue) of OTS964 in the assayed culture (*n*) at day 7 (A–F) and at day 14 (G–I). When 300 nM of OTS964 was administered in a generation (*n*–1), then an assay was conducted in the absence (orange) and the presence (red) of OTS964 in the following assayed generations (*n*). When 300 nM of OTS964 was once administered at a generation (*n*–2) of GS clones, and then a population was reconstructed (*n*–1) in the absence of OTS964. The reconstructed population was again dissociated and assayed at the following generations (*n*) in the absence or presence (green or magenta, respectively) of OTS964.

A

Size of Populations

U87, day 7

OTS964 (nM)	0_0_300	0_300_0	0_300_300	300_0_0	300_0_300
0_0_0	**	ns	**	**	**
0_0_300		**	**	**	ns
0_300_0			ns	ns	**
0_300_300				ns	**
300_0_0					** (**)

U251, day 7

OTS964 (nM)	0_0_300	0_300_0	0_300_300	300_0_0	300_0_300
0_0_0	**	ns	**	**	**
0_0_300		**	ns	**	ns
0_300_0			ns	ns	**
0_300_300				ns	*
300_0_0					** (**)

U251, day 14

OTS964 (nM)	0_0_300	0_300_0	0_300_300	300_0_0	300_0_300
0_0_0	**	ns	**	**	**
0_0_300		**	**	ns	ns
0_300_0			**	**	**
0_300_300				ns	**
300_0_0					ns (**)

Number of Clones

U87, day 7

OTS964 (nM)	0_0_300	0_300_0	0_300_300	300_0_0	300_0_300
0_0_0	**	**	**	**	**
0_0_300		**	**	**	ns
0_300_0			ns	ns	**
0_300_300				ns	**
300_0_0					** (**)

U251, day 7

OTS964 (nM)	0_0_300	0_300_0	0_300_300	300_0_0	300_0_300
0_0_0	**	**	**	ns	**
0_0_300		**	**	**	ns
0_300_0			ns	ns	**
0_300_300				ns	**
300_0_0					** (**)

U251, day 14

OTS964 (nM)	0_0_300	0_300_0	0_300_300	300_0_0	300_0_300
0_0_0	**	*	**	ns	**
0_0_300		**	**	**	ns
0_300_0			ns	ns	**
0_300_300				ns	**
300_0_0					** (**)

Size of Clones

U87, day 7

OTS964 (nM)	0_0_300	0_300_0	0_300_300	300_0_0	300_0_300
0_0_0	**	*	ns	ns	**
0_0_300		**	**	**	ns
0_300_0			ns	**	**
0_300_300				ns	**
300_0_0					** (**)

U251, day 7

OTS964 (nM)	0_0_300	0_300_0	0_300_300	300_0_0	300_0_300
0_0_0	**	ns	ns	*	**
0_0_300		**	ns	ns	ns
0_300_0			ns	*	**
0_300_300				ns	ns
300_0_0					** (**)

U251, day 14

OTS964 (nM)	0_0_300	0_300_0	0_300_300	300_0_0	300_0_300
0_0_0	**	ns	ns	**	**
0_0_300		**	*	ns	ns
0_300_0			**	**	**
0_300_300				ns	**
300_0_0					ns (*)

B

Size of Populations (>1)

U87, day 7

OTS964 (nM)	0_0_300	0_300_0	0_300_300	300_0_0	300_0_300
0_0_0	**	ns	**	**	**
0_0_300		**	**	**	ns
0_300_0			ns	ns	**
0_300_300				ns	**
300_0_0					** (**)

U251, day 7

OTS964 (nM)	0_0_300	0_300_0	0_300_300	300_0_0	300_0_300
0_0_0	**	ns	**	**	**
0_0_300		**	ns	ns	ns
0_300_0			ns	ns	**
0_300_300				ns	ns
300_0_0					* (**)

U251, day 14

OTS964 (nM)	0_0_300	0_300_0	0_300_300	300_0_0	300_0_300
0_0_0	**	ns	**	**	**
0_0_300		**	**	ns	ns
0_300_0			**	**	**
0_300_300				ns	*
300_0_0					ns (**)

Number of Clones (>1)

U87, day 7

OTS964 (nM)	0_0_300	0_300_0	0_300_300	300_0_0	300_0_300
0_0_0	**	**	**	**	**
0_0_300		**	**	**	ns
0_300_0			ns	ns	**
0_300_300				ns	**
300_0_0					** (**)

U251, day 7

OTS964 (nM)	0_0_300	0_300_0	0_300_300	300_0_0	300_0_300
0_0_0	**	**	**	**	**
0_0_300		**	**	**	ns
0_300_0			ns	ns	**
0_300_300				ns	**
300_0_0					** (**)

U251, day 14

OTS964 (nM)	0_0_300	0_300_0	0_300_300	300_0_0	300_0_300
0_0_0	**	ns	**	**	**
0_0_300		**	**	ns	ns
0_300_0			ns	ns	**
0_300_300				ns	**
300_0_0					ns (**)

Size of Clones (>1)

U87, day 7

OTS964 (nM)	0_0_300	0_300_0	0_300_300	300_0_0	300_0_300
0_0_0	**	ns	ns	ns	**
0_0_300		**	**	**	ns
0_300_0			*	ns	**
0_300_300				ns	**
300_0_0					** (**)

U251, day 7

OTS964 (nM)	0_0_300	0_300_0	0_300_300	300_0_0	300_0_300
0_0_0	**	ns	ns	ns	**
0_0_300		**	ns	ns	ns
0_300_0			*	**	**
0_300_300				ns	ns
300_0_0					ns (**)

U251, day 14

OTS964 (nM)	0_0_300	0_300_0	0_300_300	300_0_0	300_0_300
0_0_0	**	*	ns	**	**
0_0_300		**	ns	ns	ns
0_300_0			**	**	**
0_300_300				ns	**
300_0_0					ns (**)

Supplementary Figure 6: Statistical analysis for Figure 4 and Supplementary Figure 5. (**A**, **B**) The tables show the results of One-way ANOVA analysis where data of different OTS964-administration paradigms were compared in the differences in the size of the populations (left), the number of clones (middle) and the size of clones (right). The data came from U87- and U251-derived GS clones at day 7 and 14 as shown in the Figure 4 (A) and Supplementary Figure 5 (B), respectively. The asterisks in the parentheses indicate the results of the comparison by Student *t*-test (300_0_0 for the self-renewed versus 300_0_300 for the re-sensitized GS populations; they are in line with the diagrams shown in Supplementary Figure 4B).
